# One-step integrated coronary–carotid–cerebral computed tomography angiography to evaluate cardiovascular and cerebrovascular atherosclerosis

**DOI:** 10.1186/s12872-023-03343-3

**Published:** 2023-07-21

**Authors:** Shurong Liu, Zhen Zhang, Baoliang Liu, Shanshan Zhou, Jianan Xie, Ruijuan Han, Sun Kai

**Affiliations:** 1 Medical imaging research institute of Longgang, The Third People’s Hospital of Longgang District, Shenzhen, China; 2 Shenzhen Clinical Medical School, Guangzhou University of Chinese Medicine, Shenzhen, Guangdong China; 3 Department of Cardiology, The People’s Hospital of Long Gang District, Shenzhen, China; 4 Guangdong Key Laboratory for Biomedical Measurements and Ultrasound Imaging, School of Biomedical Engineering, Shenzhen University Health Science Center, Shenzhen, 518060 China; 5 Joint Laboratory of South China Hospital of Shenzhen University and Third People’s Hospital of Longgang District, South China Hospital of Shenzhen University, Shenzhen, China

**Keywords:** Atherosclerosis, Carotid artery, Cerebrovascular disease, Computed tomography angiography, Coronary artery disease, Plaque

## Abstract

**Purpose:**

This study aims to develop a low-radiation dose, one-step integrated coronary–carotid–cerebral computed tomography angiography (ICCC-CTA) technique to analyze the relationship between cardiovascular and cerebrovascular atherosclerosis and evaluate the risk factors of plaque to provide an early-stage treatment to patients and reduce vascular events.

**Methods:**

A total of 300 consecutive asymptomatic patients with cardiovascular risk factors who underwent ICCC-CTA were enrolled in this prospective study. The association between coronary and carotid-cerebrovascular atherosclerosis was assessed. The primary cardiovascular risk factors for various plaque types in cardiovascular or cerebrovascular disease were evaluated using multivariate analysis.

**Results:**

Among 300 patients, 189 (63%) had plaques in their coronary and cerebral arteries. The presence of calcified and mixed plaques in the carotid-cerebral and coronary arteries was strongly correlated (χ^2^ = 20.71, *P* = 0.001; χ^2^ = 8.96, *P* = 0.003, respectively). Multivariate logistic regression analysis revealed that abnormal blood glucose [OR = 1.44, 95% CI 0.12–0.62, *P* = 0.01] and abnormal total cholesterol [OR = 1.28, 95% CI 0.07–0.46, *P* = 0.01] are risk factors in all the models in the coronary artery, non-calcified plaque group. Abnormal blood glucose [OR = 1.43, 95% CI 0.11–0.61, *P* = 0.01] and abnormal systolic blood pressure [OR = 1.02, 95% CI 0.01–0.04, *P* = 0.02] are risk factors in all the models in the coronary artery calcified plaque group. Abnormal blood glucose level [OR = 1.44, 95% CI = 0.12–0.62, *P* = 0.01] was only a risk factor in the non-calcified plaque carotid–cerebral artery group.

**Conclusions:**

We confirm that elevated blood glucose and total cholesterol levels are associated with coronary and carotid-cerebrovascular plaques using the novel one-step low dose cerebral-carotid-cardiac CTA technique. These findings will provide insights for further studies focusing on developing low-radiation dose one-step ICCC-CTA to screen cardiovascular/cerebrovascular plaques in general population with cardiovascular risk factors.

**Advances in knowledge:**

We developed a low–radiation dose, one-step ICCC-CTA technique to detect cardiovascular and cerebrovascular atherosclerosis. We evaluated the risk factors for plaque burden for the early treatment and reduction of vascular events. These findings supported the development of low–radiation dose one-step ICCC-CTA to screen for cardiovascular/cerebrovascular disease in general population with cardiovascular risk factors.

## Introduction

Atherosclerosis is the major cause of ischemic stroke and acute coronary syndrome [1, 2]. Atherosclerosis commonly affects multiple vascular beds. Different arterial beds typically share the same risk of stenosis during atherosclerosis formation [3, 4]. Previous studies [5, 6] demonstrated that plaque accumulation in the coronary and carotid arteries had the same genetic basis, and a association existed between carotid and coronary diseases because they shared similar risk factors [7–9]. Thus, identifying the risk factors for, and the association between, coronary and carotid/cerebrovascular atherosclerosis to enable early treatment and reduce vascular events is highly significant.

Cardiac computed tomography angiography (CTA) is used to study plaques and plaque components due to its higher spatial and contrast resolution. Recent studies have revealed that a comprehensive CTA protocol is an attractive tool that can be used to evaluate coronary, carotid, and cerebral artery atherosclerosis simultaneously [12–14]. We previously reported using a low radiation dose and a dual-source computed tomography (CT) system with good image quality and high diagnostic accuracy to evaluate coronary, carotid, and cerebral artery stenosis simultaneously in 2015 [12].

The present study aims to advance our previous investigation by developing the ICCC-CTA technique to analyze the association between cardiovascular and cerebrovascular atherosclerosis and evaluating risk factors for plaque burden to enable early intervention and reduce the burden of cardiovascular/cerebrovascular disease.

## Materials and methods

### Ethical statement

The clinical study was approved by the ethics committee of the Inner Mongolia Medical University of China (No. YKD2015061) and The Third People’s Hospital of Longgang District, Shen Zhen. All procedures were by the Declaration of Helsinki. All patients provided their written informed consent to participate in the study. The data did not contain any information that could identify patients.

### Study participants

**Fig. 1:**
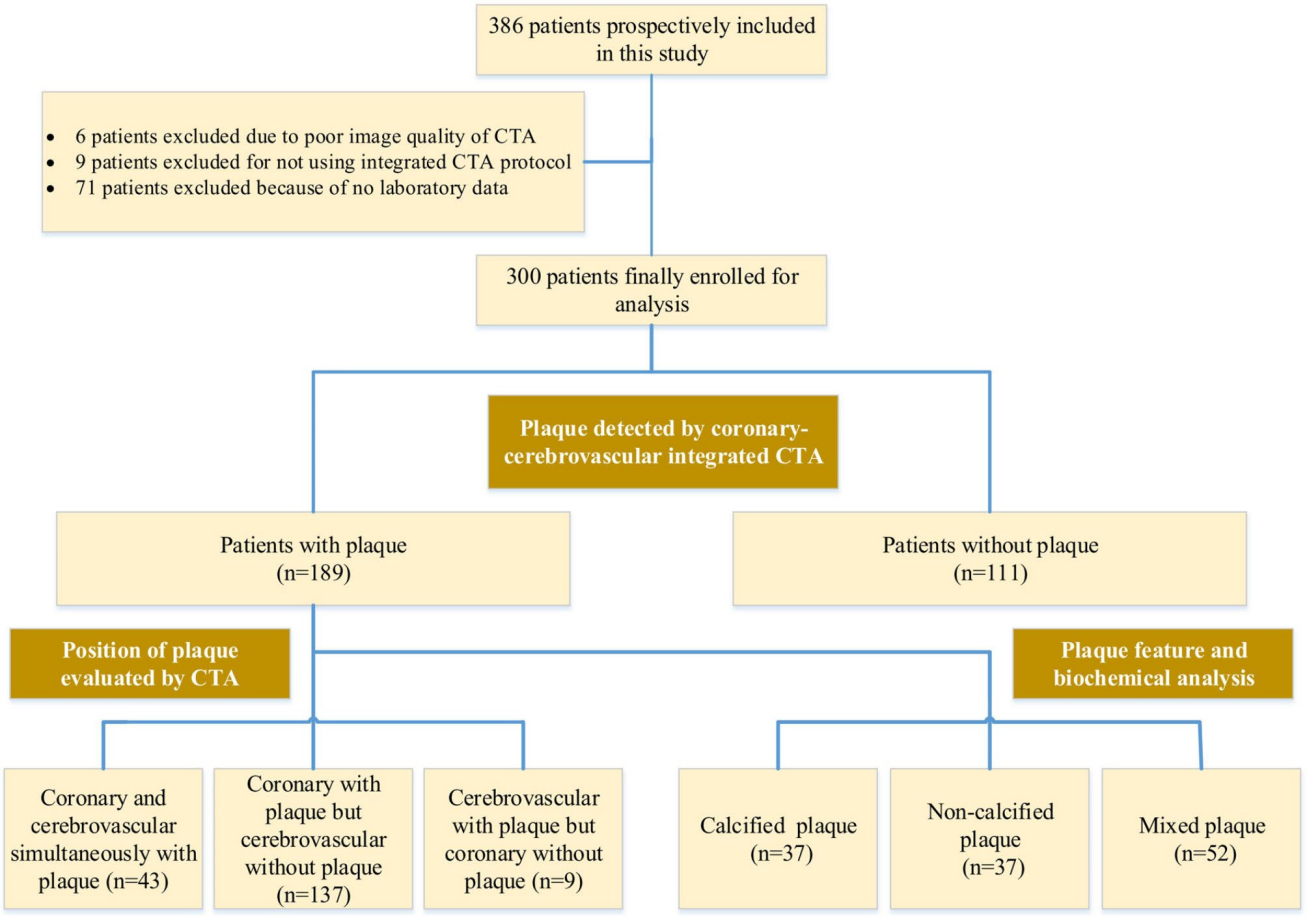
Flow chart depicting results of patient inclusion

A total of 386 consecutive asymptomatic patients with cardiovascular risk factors were enrolled and underwent ICCC-CTA between January 2015 and December 2017. Patients with iodinated contrast allergies, renal disease with a serum creatinine concentration of 1.5 mg/mL, pregnancy, and irregular heart rate were excluded. After exclusion, 300 patients remained in the study. Figure 1 displays the flow chart depicting patient inclusion results.

All patients provided baseline demographic data. The clinical and laboratory data, including sex, age, height, weight, smoking history, family history, history of diabetes, blood pressure, hyperlipidemia, total cholesterol concentration, high-density lipoprotein cholesterol concentration, low-density lipoprotein cholesterol concentration, triglyceride concentration, and creatinine concentration, were obtained.

### Cardiovascular risk factor assessment

The primary cardiovascular risk factors included diabetes mellitus, hypertension, dyslipidemia, smoking, alcohol consumption, and family history of coronary artery disease (CAD). Diabetes mellitus was defined as a fasting glucose concentration of ≥ 6.1 mmol/L, a non-fasting glucose concentration of ≥ 11.1 mmol/L, or hypoglycemic therapy administration (insulin, oral hypoglycemic therapy, or dietary advice). Hypertension was considered if the patient had a previously established diagnosis, a systolic blood pressure of ≥ 140 mm Hg, a diastolic blood pressure of ≥ 90 mm Hg, or an intake of antihypertensive medications. Dyslipidemia was defined according to the patient’s medical history or based on their current use of lipid-lowering drugs. The smoking status and alcohol consumption were ascertained by the medical history.

### Acquisition of CT data

**Fig. 2:**
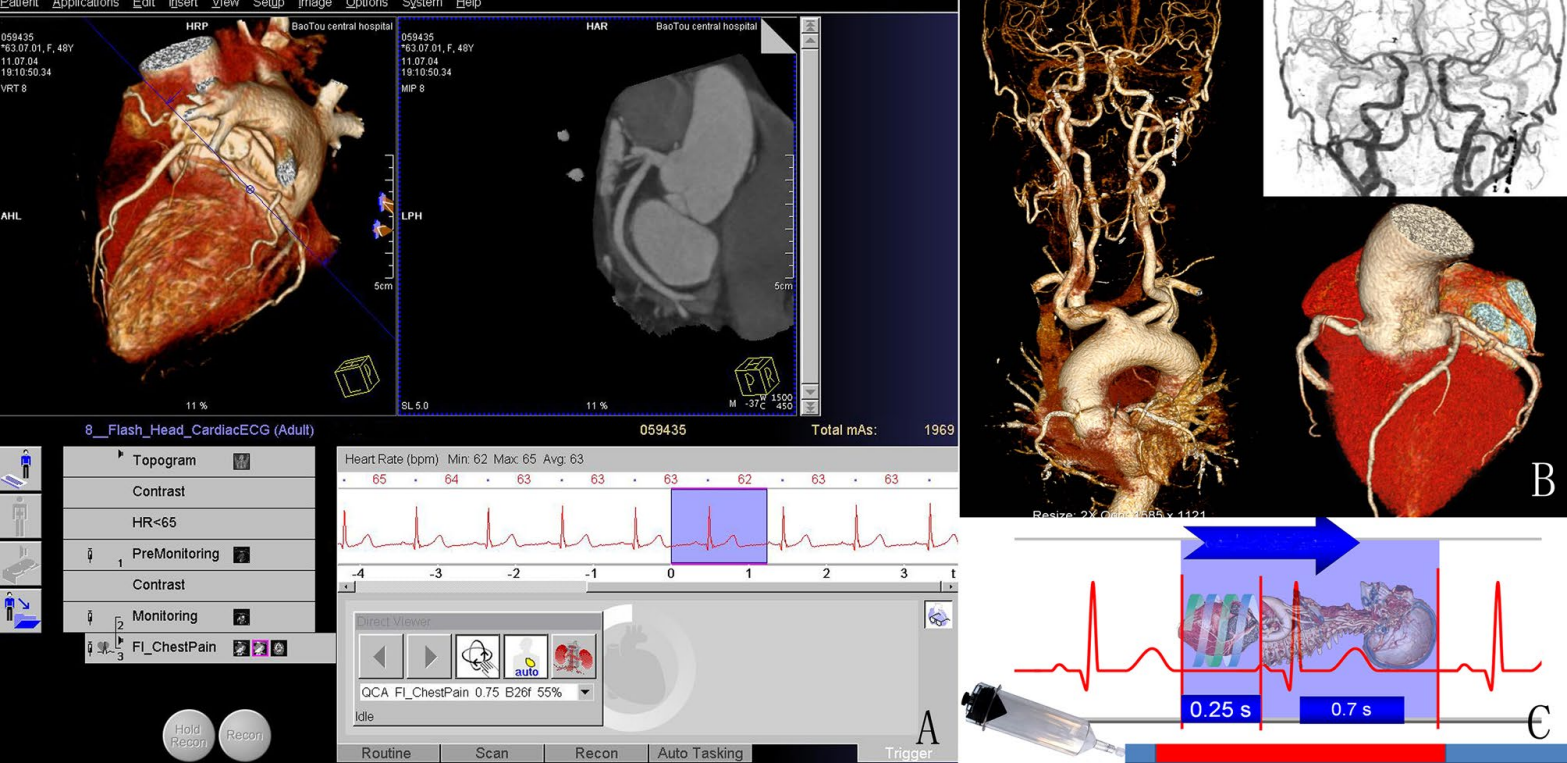
Operator interface **(A)** and typical image **(B)** of the high-pitch protocol for one-step ICCC-CTA. Scan direction and the relationship between contrast agent injection time and scan time with the ICCC-CTA protocol (C). ICCC-CTA, Integrated coronary–carotid–cerebral computed tomography angiography

A single 0.8 mg dose of nitroglycerin aerosol (Yixinbao, Shandong Jingwei Pharma, China) was administrated to the patient 3 min before scanning. The low-radiation dose ICCC-CTA scans were performed using a third-generation dual-source CT scanner (SOMATOM Force; Siemens Healthcare, Forchheim, Germany) equipped with a fully integrated circuit detector system (Stellar Infinity, Siemens). The contrast agent (Omnipaque 350 mg/mL; GE Healthcare, USA) was injected intravenously via the antecubital vein using a power injector (Missouri-XD2001; Ulrich GmbH & Co. KG, Germany) with a 20G needle. The operator interface and protocol of ICCC-CTA are depicted in Fig. 2; Table 1, respectively.

**Table 1:** The parameters of CT scanning and injection of contrast agent

CT scanning parameters		Parameters of contrast agent injection	
Acquisition protocol mode	Turbo high-pitch mode	Needle-gauge	20-gauge
Detector collimation	2 *192* 0.6 mm	Contrast agent	Omnipaque
Rotation time	0.25 s	Concentration of contrast agent	350mgI/ml
Pitch	3.2 pitch	Contrast volume	45 ml
Tube voltage	Automated tube voltage adaptation	Contrast flow rate	4.5 ml/s
Tube current	Automated tube current modulation	Saline solution volume	45 ml
Scan area	From the diaphragm to the vertex	Saline solution flow rate	4.5 ml/s
Scan direction	Caudo-cranial direction	Intravenous bolus technique	Bolus-tracking
Slice width	0.6 mm	Signal attenuation threshold	100Hu
Scan time	0.78 ± 0.12 s	Region of interest (ROI)	Ascending aorta
Effective radiation dose	1.48 ± 0.33(0.79–2.77)mSv	Heart rate	78.50 ± 21.62(54 ~ 285)bpm
Image acquisition phase	30% (HR > 65 bpm) or 60% (HR ≦ 65 bpm) of the RR interval on ECG	Delay time	8 s

Abbreviations: HR = heart rate; bpm = beats per minute;ECG = electrocardiogram

### Image reconstruction

The axial images were transferred to the workstation (Syngo. Via CTA, Siemens Healthcare). All studies were reconstructed using an advanced modeled iterative reconstruction at a strength of 3, using a medium sharp convolution kernel (Bv36), a 0.6-mm section thickness, and an increment of 0.4 mm. Curved planar reformatting (CPR) (thickness 8.0 mm), maximum intensity projection (thickness 10.0 mm), multiplanar reformatting, and volume rendering (VR) were used to evaluate the carotid, cerebrovascular, and coronary arteries.

### CT angiography analysis

The coronary tree was subdivided for segment-based analysis according to the American Heart Association standards [15]. Segments 1–4 were designated the right coronary artery, while segment 5 was designated the left main coronary artery. The left anterior descending artery comprised segments 6–10, whereas the left circumflex artery comprised segments 11–15. An intermediate artery was defined as segment 16. According to the criteria of the North American Symptomatic Carotid Endarterectomy Trial, the bilateral carotid and cerebrovascular arteries were divided into 40 segments [16]: common carotid arteries (two sides, one segment per vessel), carotid bifurcation (two sides, one segment per vessel), external carotid arteries (two sides, one segment per vessel), internal carotid arteries (two sides, seven segments per vessel), vertebral arteries (two sides, two segments per vessel), basilar artery (two sides, one segment per vessel), anterior cerebral artery (two sides, two segments per vessel), middle cerebral artery (two sides, two segments per vessel), posterior cerebral artery (two sides, two segments per vessel), anterior communicating artery (one side, one segment per vessel), and posterior communicating artery (two sides, one segment per vessel). The plaque characteristics in each vascular segment were identified and evaluated as calcified, non-calcified, or mixed as previously described [11, 17]. The coronary atherosclerotic lesions were quantified for stenosis using quantitative analysis. The degree of stenosis was measured as the ratio between the luminal diameters of the segments exhibiting obstruction and the luminal diameter of the most normal-appearing site immediately proximal to the plaque in multiplanar curved reformatted images. The evaluable coronary artery segments were assessed for stenosis using six predefined categories: 0%, 1–24%, 25–49%, 50–69%, 70–99%, or 100% (total occlusion) according to the CAD Reporting and Data System (CAD-RADS) [18].

### Estimation of radiation dose

The volume CT dose index and dose–length product (DLP) were recorded automatically at the end of each scan. The effective radiation dose (in mSv) was estimated by multiplying the DLP with a conversion factor (*k* = 0.026 mSv·mGy^− 1^ cm^− 1^ for cardiovascular imaging and *k* = 0.0031 mSv mGy^− 1^ cm^− 1^ for the head and neck) according to the previous studies [19, 20].

### Statistical analysis

The continuous variables were presented as mean ± standard deviation or median (interquartile range) if appropriate (non-normal distribution). The categorical variables were displayed as numbers (%). The Student *t*-test for independent samples was used to compare normally distributed continuous variables. When the variable distribution was non-normal, the Wilcoxon rank-sum test was used for independent samples. The chi-squared and Fisher’s exact tests were used to compare categorical and skewed variables. The influence of selected patient characteristics, plaque risk factors, and scan characteristics on plaque burden was visualized using odds ratio (OR) with 95% confidence interval (CI) in a multivariate logistic regression model. A *P*-value of < 0.05 indicated a statistically significant difference. All statistical analyses were performed using IBM SPSS Statistics version 21.0 (NY, USA) or GraphPad Prism version 4.02 (CA, USA) and Quickcalcs 2014.

## Results

This study enrolled 386 consecutive patients to undergo ICCC-CTA. After exclusion, the study included 300 patients (77.7%). Exclusion criteria included poor image quality on CTA (*n* = 6), failure to use the ICCC-CTA protocol (*n* = 9), and a lack of laboratory data (*n* = 71). Figure 1 presents that 72.5% of 189 patients with plaques had only coronary plaques, 22.8% had only cerebrovascular plaques, and 4.8% had plaques in both territories. In this study, the mean age of patients was 56 ± 10 years (range, 37–92 years), with 66% (199 out of 300) male. The mean heart rate during CTA was 74 ± 11 beats per minute (bpm) (range, 40–128 bpm). The average DLP for the ICCC-CTA was 138.57 ± 31.6 mGy cm with a calculated corresponding effective radiation dose of 1.48 ± 0.33 mSv. Tables 1 and 2 present the patient’s baseline and clinical characteristics.

**Table 2:** Baseline and characteristics of the patient population

	All Population (n = 300)	Patients with plaque (n = 189)	Patients without plaque (n = 111)	*p* value
Clinical characteristics				
Gender,%(Male)	66.33(199)	70.37(133)	59.45(66)	0.059
Age,years	56 ± 10	58 ± 10	53 ± 9	0.001
Mean Heart rate (bpm)	74 ± 11	74 ± 12	73 ± 10	0.868
Hypertension,%(n)	10.3(81)	35.4(67)	12.6(14)	0.001
Diabetes,%(n)	7.4(58)	28.6(54)	3.6(4)	0.001
SBP, mmHg	135.48 ± 11.54	135.60 ± 18.31	134.30 ± 10.86	0.495
DBP,mmHg	81.32 ± 8.95	81.73 ± 8.80	79.48 ± 7.65	0.028
BMI,kg/m^2^	22.42 ± 2.29	22.62 ± 2.59	22.28 ± 1.92	0.224
Education level,%(n)				0.593
< 6years	0.6(5)	2.1(4)	0.9(1)	
6-12years	17.1(135)	43.4(82)	47.7(53)	
> 12years	20.3(160)	54.5(103)	51.4(57)	
Health habits				
Current cigarette user,%(n)	23.9(188)	65.6(124)	57.7(64)	0.169
Current alcohol user,%(n)	26.8(211)	73.5(139)	64.9(72)	0.118
Physical activity,%(n)				0.976
Active	37.1(292)	97.4(184)	97.3(108)	
Not Active	1(8)	2.6(5)	2.7(3)	
Laboratory data				
FG,mmol/L	5.58 ± 1.14	5.58 ± 2.50	4.76 ± 1.31	0.001
TC,mmol/L	1.69 ± 0.98	1.82 ± 1.14	1.66 ± 0.94	0.004
LDL-C,mmol/L	2.48 ± 0.79	2.55 ± 0.90	2.33 ± 0.59	0.04
HDL-C,mmol/L	1.68 ± 0.75	1.73 ± 0.77	1.56 ± 0.93	0.119
Uric acid,umol	6.04 ± 2.15	6.02 ± 2.72	6.26 ± 1.68	0.442
Creatinine,umol/L	65.92 ± 20.39	67.55 ± 26.88	66.33 ± 13.53	0.683

Abbreviations: FG = fasting glucose;TC = total cholesterol; HDL-C = high-density lipoprotein cholesterol; LDL-C = low-density lipoprotein cholesterol

### Association between coexisting plaques in coronary, carotid, and cerebral arteries

**Fig. 3:**
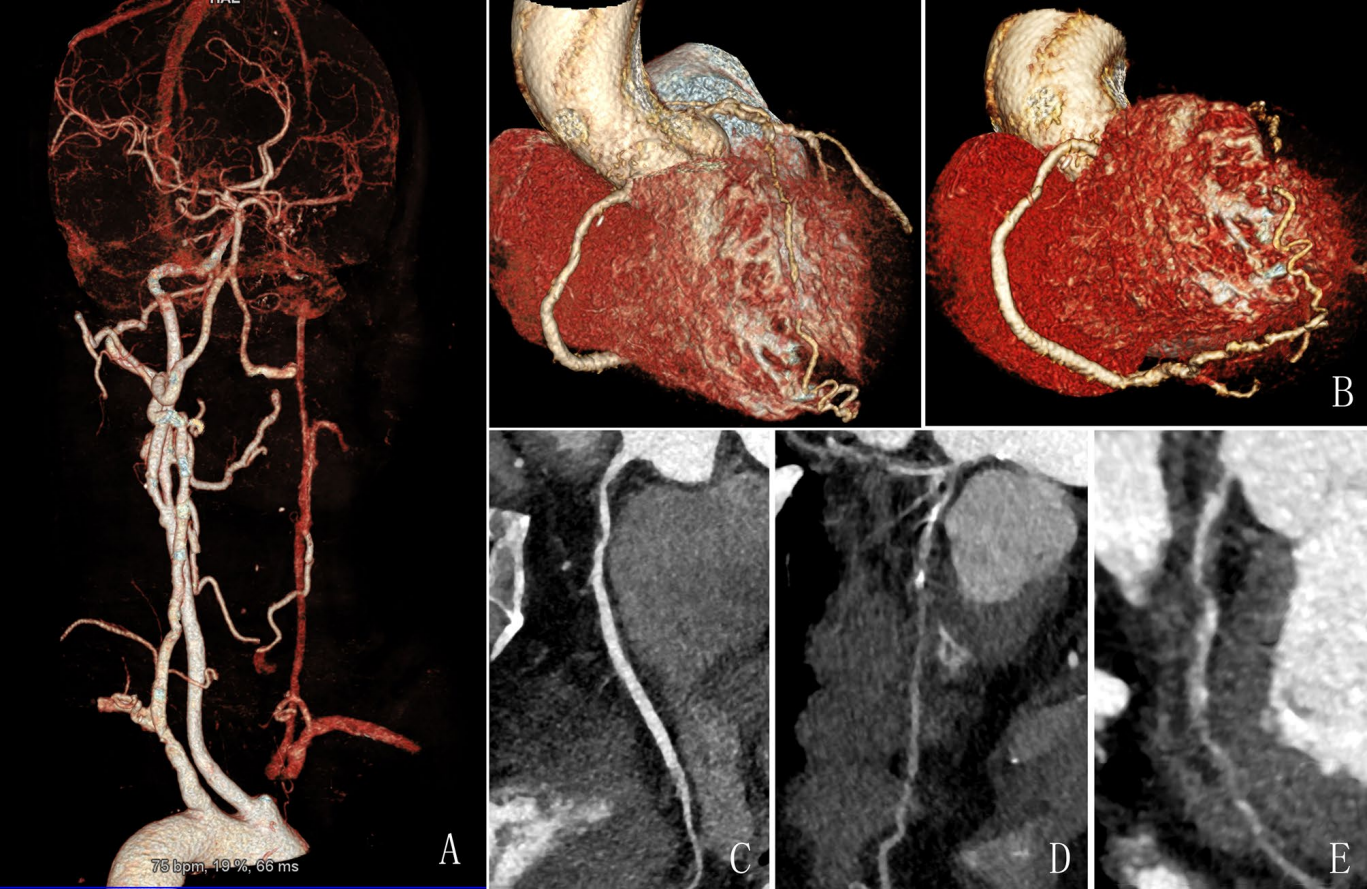
A 50-year-old male patient with progressive leg weakness, numbness, and dizziness for 2 days. ICCC-CTA detected coexisting plaques in the carotid [total occlusion in the proximal brachiocephalic trunk **(A)**] and coronary arteries [stenosis in RCA **(C)**, LAD (**B** and **D**), and LCx **(E)** arteries]. ICCC-CTA, Integrated coronary–carotid–cerebral computed tomography angiography; LAD, left anterior descending; LCx, left circumflex; RCA, right coronary artery; VR, volume rendering

Among 300 patients, 189 (63%) presented with plaques, whereas 111 patients (37%) did not present with plaques in the coronary and cerebral arteries. Of these plaques, 180 (60%) were in the coronary artery, and 52 (17.3%) were in the carotid and cerebral arteries. A total of 43 patients (14.3%) had coexisting plaques in carotid or cerebral arteries. A association was discovered between the constituent ratio of coronary plaques and carotid–cerebral artery plaques (*χ*^2^ = 14.22, *P* = 0.001, Table 3). A strong association was observed between the presence of calcified and mixed plaques in the carotid–cerebral and coronary arteries (*χ*^2^ = 20.71, *P* = 0.001; *χ*^2^ = 8.96, *P* = 0.003, respectively), while no association was discovered between non-calcified plaques in the coronary and the carotid–cerebral arteries (*χ*^2^ = 2.93, *P* = 0.087, Table 4). A significant association was observed between coronary and cerebrovascular plaques. However, 76% of patients with coronary plaques had no cerebrovascular plaques. Figure 3 presents a patient with coexisting plaques in the carotid, and coronary arteries detected using one-step ICCC-CTA.

**Table 3:** The constituent ratio of plaque position in coronary and carotid-cerebrovascular

		Plaque in coronary		total	χ^2^	*p* value
		(+)	(-)			
**Plaque in carotid-cerebrovascular**	(+)	43	9	52	14.22	0.001
	(-)	137	111	248		
**total**		180	120	300		

**Table 4:** The constituent ratio of various plaque types in coronary and carotid-cerebrovascular

	plaque features		coronary		χ^2^	*p* value
			(+)	(-)		
	calcified	(+)	22	15	20.71	0.001
		(-)	62	201		
**carotid-cerebrovascular**	non-calcified	(+)	18	19	2.93	0.087
		(-)	90	173		
	mixed	(+)	25	27	8.96	0.003
		(-)	67	181		

### Multivariate analysis of cardiovascular risk factors

**Fig. 4:**
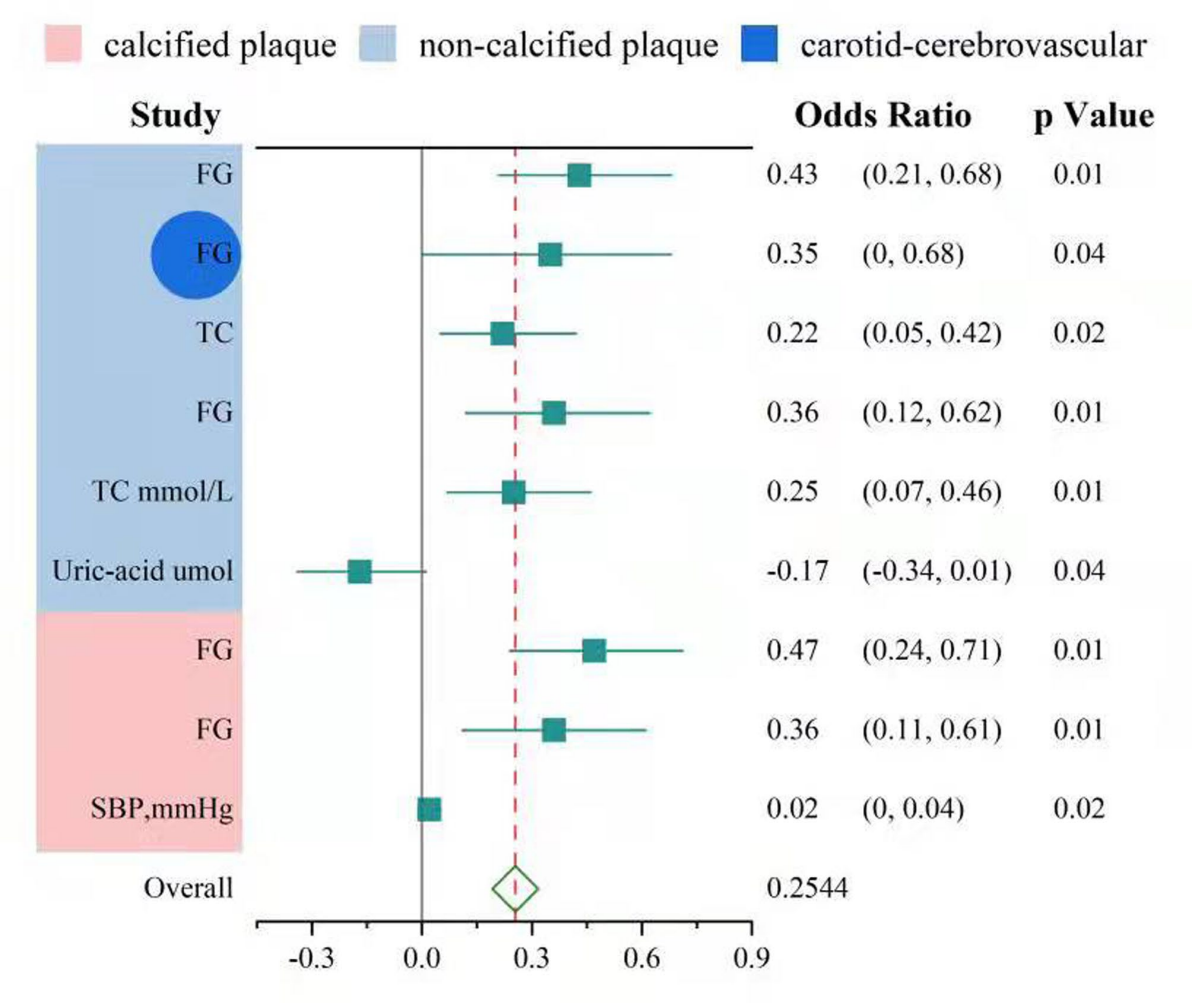
Multivariate logistic regression analysis of cardiovascular and cerebrovascular risk factors for non-calcified (graph A) and calcified (graph B) plaque burden

**Fig. 5:**
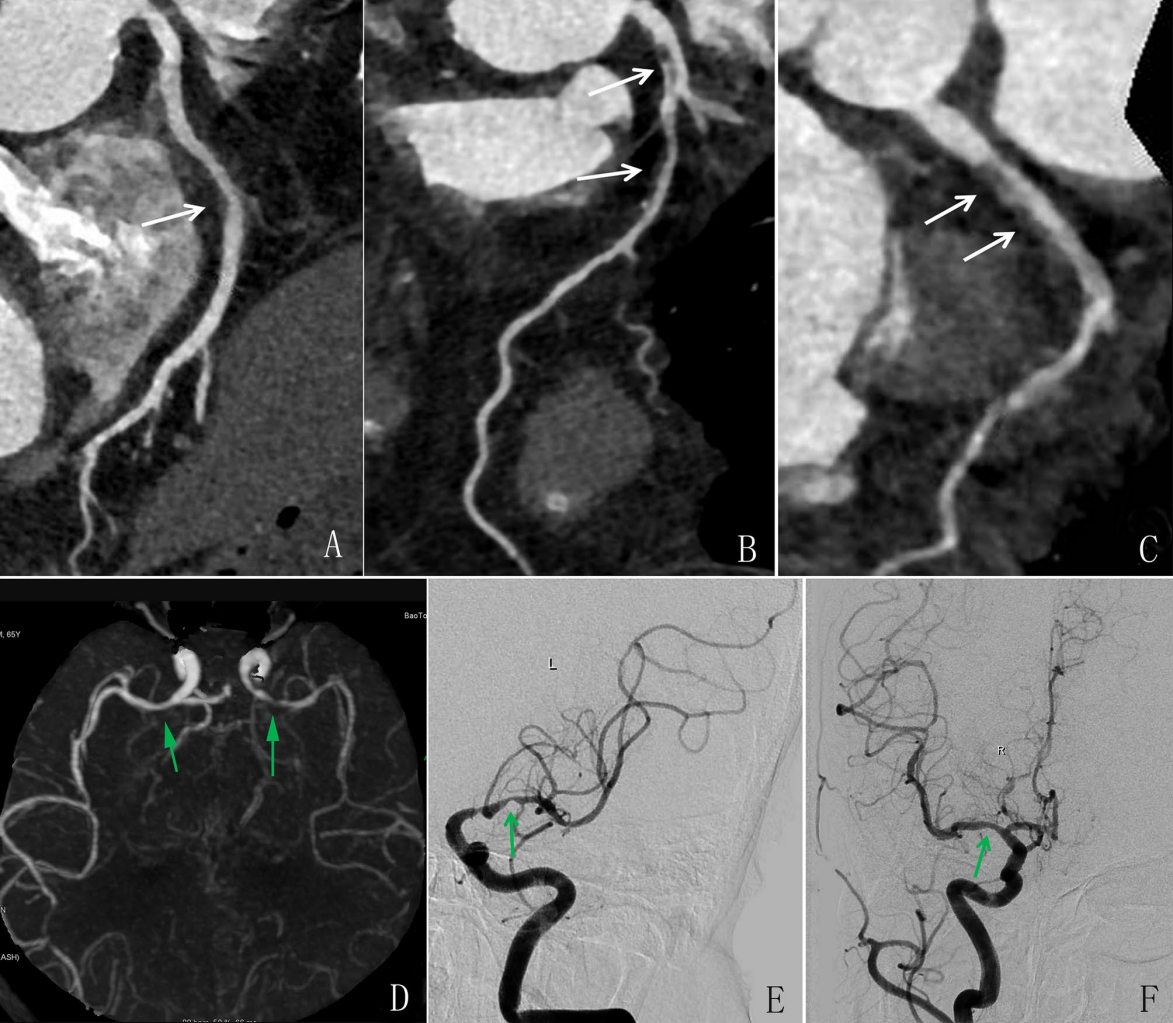
ICCC-CTA in a 72-year-old female patient with a 10-year history of diabetes. CPR of the RCA **(A)**, LAD artery **(B)**, and LCx artery **(C)** showed mild-to-severe stenosis with non-calcified plaques (*white arrow*). MIP of the cerebrovascular CTA showed mild stenosis in the M1 segment of the right MCA and severe stenosis in the M1 segment of the left MCA (*green arrow*). Invasive angiography confirmed stenosis in the left MCA (E) and right MCA (F) (*green arrow*). CPR, Curved planar reformation; ICCC-CTA, integrated coronary–carotid–cerebral computed tomography angiography; LAD, left anterior descending; LCx, left circumflex; MCA, middle cerebral artery; MIP, maximum intensity projection; RCA, right coronary artery

Figure 4 depicts a statistical graph of the multivariate logistic regression analysis of non-calcified and calcified plaques for cardio-cerebrovascular risk factors. Multivariate analysis revealed that the primary cardiovascular risk factors, including FG, age, and TC, were associated with various plaque types in the coronary and carotid–cerebral arteries (Table 5–6). Abnormal blood glucose [OR = 1.44, 95% CI 0.12–0.62, *P* = 0.01] and abnormal total cholesterol [OR = 1.28, 95% CI 0.07–0.46, *P* = 0.01] are risk factors in all the models of non-calcified plaque group. Abnormal blood glucose [OR = 1.44, 95% CI 0.12–0.62, *P* = 0.01] is a risk factor only in model 1 of the carotid-cerebral artery non-calcified plaque group. However, abnormal blood glucose [OR = 1.43, 95% CI 0.11–0.61, *P* = 0.01] and abnormal systolic blood pressure [OR = 1.02, 95% CI 0.01–0.04, *P* = 0.02] are risk factors in all the models in the coronary artery of calcified plaque group. Figure 5 displays a typical image of a diabetic patient with FG associated with plaques in the coronary and cerebral arteries.

**Table 5:** Association between non-calcified plaque burden with cardiovascular and cerebrovascular risk factors

Variables	Coronary				carotid-cerebrovascular			
	β	OR	95%CI	*p* value	β	OR	95%CI	*p* value
Model 1*								
FG	0.43	1.55	0.21–0.68	0.00	0.35	1.41	0.00-0.68	0.04
TG	-0.04	0.96	-0.31-0.23	0.78	-0.28	0.76	-0.95-0.23	0.35
TC	0.22	1.25	0.05–0.42	0.02	-0.02	0.98	-0.30-0.20	0.88
**Model 2** ^**#**^								
FG	0.37	1.45	0.13–0.63	0.00	0.28	1.33	-0.08-0.65	0.13
TG	-0.05	0.95	-0.33-0.22	0.71	-0.26	0.77	-0.95-0.27	0.40
TC	0.23	1.26	0.06–0.44	0.02	-0.01	0.99	-0.32-0.22	0.94
HDL-C	0.02	1.02	-0.39-0.39	0.93	-0.14	0.87	-0.96-0.39	0.69
LDL-C	-0.04	0.96	-0.45-0.36	0.83	0.13	1.14	-0.56-0.82	0.71
SBP	0.02	1.02	0.00-0.04	0.06	0.01	1.01	-0.02-0.04	0.45
DBP	-0.02	0.98	-0.06-0.02	0.31	0.03	1.03	-0.03-0.09	0.25
**Model 3^**								
**FG**	0.36	1.44	0.12–0.62	0.00	0.28	1.32	-0.09-0.65	0.13
TG,mmol/L	-0.09	0.91	-0.38-0.19	0.53	-0.26	0.77	-0.95-0.28	0.41
TC,mmol/L	0.25	1.28	0.07–0.46	0.01	-0.01	0.99	-0.32-0.23	0.96
HDL-C,mmol/L	0.10	1.11	-0.30-0.50	0.59	-0.16	0.85	-1.00-0.39	0.64
LDL-C,mmol/L	0.02	1.02	-0.40-0.43	0.94	0.12	1.13	-0.57-0.82	0.73
SBP, mmHg	0.02	1.02	0.00-0.04	0.09	0.01	1.01	-0.02-0.04	0.44
DBP,mmHg	-0.02	0.98	-0.06-0.01	0.21	0.03	1.03	-0.03-0.09	0.27
Uric acid,umol	-0.17	0.84	-0.34-0.01	0.04	0.05	1.05	-0.22-0.34	0.73
Creatinine,umol/L	0.00	1.00	-0.02-0.02	0.82	-0.01	0.99	-0.04-0.03	0.72

*crude model

#adjusted for age, gender, BMI, Uric acid and creatinine

^further adjusted for other cardiovascular and cerebrovascular risk factors

Abbreviations: FG = fasting glucose; BMI = body mass index;TC = total cholesterol; HDL-C = high-density lipoprotein cholesterol; LDL-C = low-density lipoprotein cholesterol; DBP = diastolic blood pressure

**Table 6:** Association between calcified plaque burden with cardiovascular and cerebrovascular risk factors

Variables	Coronary				carotid-cerebrovascular			
	β	OR	95%CI	*p* value	β	OR	95%CI	*p* value
Model 1*								
FG	0.47	1.59	0.24–0.71	0.00	0.22	1.24	-0.09-0.51	0.16
TG	0.09	1.10	-0.19-0.36	0.50	0.05	1.06	-0.36-0.42	0.77
TC	0.14	0.14	-0.01-0.30	0.09	-0.05	0.95	-0.29-0.14	0.66
**Model 2** ^**#**^								
FG	0.36	1.44	0.12–0.62	0.00	0.12	1.13	-0.21-0.45	0.46
TG	0.09	1.10	-0.20-0.37	0.52	0.03	1.04	-0.40-0.41	0.87
TC	0.13	1.13	-0.03-0.30	0.14	-0.08	0.93	-0.35-0.13	0.52
HDL-C	0.04	1.03	-0.40-0.40	0.90	0.20	1.22	-0.28-0.60	0.33
LDL-C	0.16	1.18	-0.25-0.58	0.44	0.08	1.08	-0.47-0.63	0.77
SBP	0.02	1.02	0.00-0.04	0.02	0.02	1.02	-0.01-0.04	0.23
DBP	-0.01	0.99	-0.05-0.03	0.69	-0.02	0.98	-0.08-0.03	0.51
**Model 3^**								
**FG**	0.36	1.43	0.11–0.61	0.00	0.12	1.13	-0.21-0.44	0.47
TG,mmol/L	0.07	1.08	-0.22-0.35	0.62	0.01	1.01	-0.42-0.39	0.94
TC,mmol/L	0.13	1.14	-0.03-0.31	0.12	-0.07	0.93	-0.34-0.13	0.55
HDL-C,mmol/L	0.03	1.03	-0.40-0.41	0.89	0.19	1.21	-0.29-0.59	0.37
LDL-C,mmol/L	0.17	1.19	-0.25-0.59	0.42	0.10	1.11	-0.46-0.65	0.72
SBP, mmHg	0.02	1.02	0.00-0.04	0.02	0.02	1.02	-0.01-0.04	0.24
DBP,mmHg	-0.01	0.99	-0.05-0.03	0.61	-0.02	0.98	-0.08-0.03	0.43
Uric acid,umol	0.00	1.01	-0.16-0.18	0.92	0.03	1.03	-0.20-0.27	0.83
Creatinine,umol/L	-0.01	0.99	-0.03-0.01	0.32	-0.02	0.99	-0.04-0.01	0.29

*crude model

#adjusted for age, gender, BMI, Uric acid and creatinine

^further adjusted for other cardiovascular and cerebrovascular risk factors

Abbreviations: FG = fasting glucose; BMI = body mass index;TC = total cholesterol; HDL-C = high-density lipoprotein cholesterol; LDL-C = low-density lipoprotein cholesterol; DBP = diastolic blood pressure

## Discussion

An easy-to-employ technical method is essential to detect cardiovascular and cerebrovascular atherosclerosis. Tognolini [14] proposed an imaging technique to assess the carotid and coronary arteries based on a combined carotid and coronary CTA protocol. However, the average radiation dose was 4.3 mSv under this protocol. The protocol required 100 mL of the contrast agent because CTA was performed with two CT acquisitions of the helical carotid CTA and prospective sequential electrocardiogram-triggered coronary CTA. Previous studies discovered that the one-step high-pitch scan protocol for combined coronary and carotid–cerebral artery CTA had a greater prognostic value for evaluating coronary and cerebral artery stenosis with a low radiation exposure of 1.42 ± 0.44 mSv and a lower volume of the contrast agent [12]. However, we performed this protocol only in patients with a stable heart rate below 65 bpm to avoid poor image quality. Furthermore, the association between cardiovascular and cerebrovascular atherosclerosis and the risk factors for plaque burden, which would enable early intervention and reduce the cardiovascular/cerebrovascular disease burden, required further investigation.

This study developed the ICCC-CTA technique to analyze the association between cardiovascular and cerebrovascular atherosclerosis. Moreover, we evaluated the risk factors for plaque burden to enable early intervention and reduce the cardiovascular and cerebrovascular disease burden. The total effective radiation dose for the coronary and carotid CTA was 1.42 ± 0.44 mSv, which was significantly lower than that reported by Tognolini et al. (4.3 mSv) [14] and Yasmin et al. (7.3 and 3.8 mSv for coronary and carotid CTA, respectively) [21].

The association between the coronary and carotid arteries lesions is well known. Calcified atherosclerotic plaques in the coronary and carotid arteries share common risk factors [22]. A moderate-to-strong association was observed between calcification in the coronary and carotid vessel beds [23]. Yasmin et al. [21] associated calcified plaques in the carotid artery with total and calcified plaques in the coronary bed, but noncalcified plaques in the coronary arteries were not. This study observed a strong association between calcified and mixed plaques in the carotid–cerebral and coronary arteries. However, we identified no association between non-calcified plaques in the coronary and the carotid–cerebral arteries. This study identified a association between coronary atherosclerotic and carotid artery plaques, supporting the previous studies that CAD was related to atherosclerotic CAD. This finding might be valuable to understand cardiovascular atherosclerosis and cerebrovascular diseases as systemic responses.

The plaque analysis using coronary CT could predict future cardiovascular events [24]. Coronary CTA is a well-established method for detecting coronary stenosis and calcified and non-calcified plaques and is a predictor of all-cause mortality. According to the concept of vulnerable plaques proposed by Naghavi et al., [25] are typically non-calcified, non-stenotic, and rich in extracellular lipids. These lesions should be identifiable via coronary CTA. Studies have indicated that carotid and coronary atherosclerosis share common risk factors, such as diabetes mellitus, hypertension, smoking, old age, high triglyceride concentrations, and low high-density lipoprotein cholesterol concentrations [26]. These risk factors have different impacts on different arterial systems. Cholesterol is particularly important in coronary atherosclerosis and hypertension in ischemic stroke, whereas smoking and diabetes are important in intermittent claudication [27, 28]. This study data associated the traditional risk factors with various plaque types in coronary and carotid–cerebral arteries, reaching statistical significance with multivariate analysis in models 1 and 3. The multivariate logistic regression analysis revealed that abnormal blood glucose and abnormal total cholesterol are risk factors in all models in the coronary artery non-calcified plaque group. Abnormal blood glucose is a risk factor only in model 1 in the carotid–cerebral artery non-calcified plaque group. In the coronary artery non-calcified plaque, a high prevalence of high-risk plaque (HRP) was detected in this population of asymptomatic Type 2 diabetes (T2D) [29]. During a nine-year follow-up in Halon’s study, the authors discovered that HRP caused most events, while events in non-HRP were rare. The risk of acute events in the study increased by the number of high-risk plaque features and the degree of stenosis [30]. These results are comparable to our study about abnormal blood glucose, although using a slightly different definition of HRP or non-calcified plaque. Carotid atherosclerotic plaque is the primary risk factor of ischemic cerebrovascular disease, vulnerable plaques (VP) rupture, secondary thrombosis, and embolism, leading to stroke. The older population was closely related to the lipid-rich necrotic core (LRNC) occurrence [31]. However, after adjusting for age, we discovered that blood glucose is abnormal [OR = 1.44, 95% CI (0.12, 0.62), *P* = 0.01] only in model 1 of the non-calcified carotid–cerebral artery plaque.

A densely calcified plaque was considered a protective factor [30]. Low-density lipoprotein cholesterol (LDL-C) has a primary role in forming atherosclerosis plaque. High serum LDL-C levels may lead to lipid deposition with macrophage accumulation [32], and a large lipid pool underneath the endothelium has a high probability of necrosis [33], accelerating endothelial injury and eventually ending with plaque rupture. These results differ from our study about systolic blood pressure. There are few studies on the risk factors of calcified plaque in the carotid–cerebral artery, and this study did not identify any significant risk factors in establishing the regression model in the carotid–cerebral artery calcified plaque.

The present study has several limitations. First, this is a single-center study, and a multi-center registry is necessary to confirm the present results. Second, this study classified plaque characteristics as calcified, non-calcified, or mixed according to the traditional classification, as previously described. Further studies are required to assess the high-risk plaque characteristics, including low-attenuation plaques, positive remodeling, napkin-ring signs, and spotty. Third, no follow-up data were available to predict cardiovascular events. Further studies over a 3–5-year follow-up period are needed to investigate coronary plaque features on cardiovascular/cerebrovascular CTA and to predict cardiovascular events. Meanwhile, the focus predominantly rests on screening healthy individuals, resulting in less emphasis on statin usage.

This study evaluated atherosclerosis using low-dose radiation one-step ICCC-CTA and demonstrated a association between cardiovascular and cerebrovascular atherosclerosis. The finding supports the future development of ICCC-CTA to screen for cardio/cerebrovascular disease in populations with cardiovascular risk factors. Abnormal blood glucose and abnormal blood lipid are the risk factors for non-coronary plaque formation (*P* < 0.05). Abnormal blood glucose and abnormal systolic blood pressure are the risk factors for calcified coronary plaque formation (*P* < 0.05).

## Conclusions

We confirm that elevated blood glucose and total cholesterol levels are associated with coronary and carotid-cerebrovascular plaques using the novel one-step low dose cerebral-carotid-cardiac CTA technique. These findings will provide insights for further studies focusing on developing low-radiation dose one-step ICCC-CTA to screen cardiovascular/cerebrovascular plaques in general population with cardiovascular risk factors.

## Data Availability

The datasets used and/or analyzed in the current study are available from the corresponding author on reasonable request.

## References

[1] Qureshi AI, Caplan LR (2014). Intracranial atherosclerosis. Lancet.

[2] Yonetsu T, Hoshino M, Lee T, Kanaji Y, Yamaguchi M, Hada M (2020). Plaque morphology assessed by optical coherence tomography in the culprit lesions of the first episode of acute myocardial infarction in patients with low low-density lipoprotein cholesterol level. J Cardiol.

[3] Razzouk L, Rockman CB, Patel MR, Guo Y, Adelman MA, Riles TS (2015). Co-existence of vascular disease in different arterial beds: peripheral artery disease and carotid artery stenosis–data from Life Line Screening((R)). Atherosclerosis.

[4] Banchhor SK, Londhe ND, Araki T, Saba L, Radeva P, Khanna NN (2018). Calcium detection, its quantification, and grayscale morphology-based risk stratification using machine learning in multimodality big data coronary and carotid scans: a review. Comput Biol Med.

[5] Craven TE, Ryu JE, Espeland MA, Kahl FR, McKinney WM, Toole JF (1990). Evaluation of the associations between carotid artery atherosclerosis and coronary artery stenosis. A case-control study. Circulation.

[6] Geroulakos G, O’Gorman DJ, Kalodiki E, Sheridan DJ, Nicolaides AN (1994). The carotid intima-media thickness as a marker of the presence of severe symptomatic coronary artery disease. Eur Heart J.

[7] Cohen GI, Aboufakher R, Bess R, Frank J, Othman M, Doan D (2013). Relationship between carotid disease on ultrasound and coronary disease on CT angiography. JACC Cardiovasc Imaging.

[8] Hamada S, Kashiwazaki D, Yamamoto S, Akioka N, Kuwayama N, Kuroda S (2018). Impact of Plaque Composition on Risk of Coronary Artery Diseases in patients with carotid artery stenosis. J Stroke Cerebrovasc Dis.

[9] Cappelletti A, Astore D, Godino C, Bellini B, Magni V, Mazzavillani M (2018). Relationship between Syntax score and prognostic localization of coronary artery lesions with conventional risk factors, plasma profile markers, and carotid atherosclerosis (CAPP Study 2). Int J Cardiol.

[10] Polak JF, Szklo M, O’Leary DH (2017). Carotid intima-media thickness score, positive coronary artery calcium score, and Incident Coronary Heart Disease: the multi-ethnic study of atherosclerosis. J Am Heart Assoc.

[11] Guaricci AI, Arcadi T, Brunetti ND, Maffei E, Montrone D, Martini C (2014). Carotid intima media thickness and coronary atherosclerosis linkage in symptomatic intermediate risk patients evaluated by coronary computed tomography angiography. Int J Cardiol.

[12] Sun K, Li K, Han R, Li W, Chen N, Yang Q (2015). Evaluation of high-pitch dual-source CT angiography for evaluation of coronary and carotid-cerebrovascular arteries. Eur J Radiol.

[13] Zhang JL, Liu BL, Zhao YM, Liang HW, Wang GK, Wan Y (2015). Combining coronary with carotid and cerebrovascular angiography using prospective ECG gating and Iterative Reconstruction with 256-slice CT. Echocardiography.

[14] Tognolini A, Arellano CS, Marfori W, Heidari G, Sayre JW, Krishnam MS (2014). Comprehensive low-dose imaging of carotid and coronary arteries with a single-injection dual-source CT angiography protocol. Clin Radiol.

[15] Austen WG, Edwards JE, Frye RL, Gensini GG, Gott VL, Griffith LS (1975). A reporting system on patients evaluated for coronary artery disease. Report of the ad Hoc Committee for Grading of Coronary Artery Disease, Council on Cardiovascular surgery, American Heart Association. Circulation.

[16] Trial C, Barnett HJM, Taylor DW, Haynes RB, Sackett DL, Peerless SJ, North American Symptomatic Carotid Endarterectomy (1991). Beneficial effect of carotid endarterectomy in symptomatic patients with high-grade carotid stenosis. N Engl J Med.

[17] Motoyama S, Kondo T, Sarai M, Sugiura A, Harigaya H, Sato T (2007). Multislice computed tomographic characteristics of coronary lesions in acute coronary syndromes. J Am Coll Cardiol.

[18] Cury RC, Abbara S, Achenbach S, Agatston A, Berman DS, Budoff MJ (2016). CAD-RADS(TM) coronary artery Disease - Reporting and Data System. An expert consensus document of the Society of Cardiovascular Computed Tomography (SCCT), the American College of Radiology (ACR) and the North American Society for Cardiovascular Imaging (NASCI). Endorsed by the American College of Cardiology. J Cardiovasc Comput Tomogr.

[19] Trattner S, Halliburton S, Thompson CM, Xu Y, Chelliah A, Jambawalikar SR (2018). Cardiac-specific Conversion factors to Estimate Radiation Effective Dose from dose-length product in computed tomography. JACC Cardiovasc Imaging.

[20] McCollough C, Edyvean S, Cody D, Geise R, Gould B. *AAPM report no. 96: the measurement, reporting, and management of radiation dose in CT—report of AAPM Task Group 23 of the Diagnostic Imaging Council CT Committee*. http://www.aapm.org/pubs/reports/RPT96.pdf.

[21] Hamirani YS, Larijani V, Isma’eel H, Pagali SR, Bach P, Karlsberg RP (2010). Association of plaque in the carotid and coronary arteries, using MDCT angiography. Atherosclerosis.

[22] Wagenknecht LE, Langefeld CD, Freedman BI, Carr JJ, Bowden DW (2007). A comparison of risk factors for calcified atherosclerotic plaque in the coronary, carotid, and abdominal aortic arteries: the diabetes heart study. Am J Epidemiol.

[23] Odink AE, van der Lugt A, Hofman A, Hunink MG, Breteler MM, Krestin GP (2007). Association between calcification in the coronary arteries, aortic arch and carotid arteries: the Rotterdam study. Atherosclerosis.

[24] Tomizawa N, Yamamoto K, Inoh S, Nojo T, Nakamura S (2018). High-risk plaque and calcification detected by coronary CT angiography to Predict Future Cardiovascular events after percutaneous coronary intervention. Acad Radiol.

[25] Naghavi M, Libby P, Falk E, Casscells SW, Litovsky S, Rumberger J (2003). From vulnerable plaque to vulnerable patient: a call for new definitions and risk assessment strategies: part I. Circulation.

[26] Alberts MJ, Bhatt DL, Mas JL, Ohman EM, Hirsch AT, Rother J (2009). Three-year follow-up and event rates in the international REduction of atherothrombosis for continued Health Registry. Eur Heart J.

[27] Kannel WB (1994). Risk factors for atherosclerotic cardiovascular outcomes in different arterial territories. J Cardiovasc Risk.

[28] Kannel WB, Wolf PA (2006). Peripheral and cerebral atherothrombosis and cardiovascular events in different vascular territories: insights from the Framingham Study. Curr Atheroscler Rep.

[29] Laurits J, Pararajasingam G, Andersen TR, Søren, Auscher HM, Sheta H, Precht J, Lambrechtsen K, Egstrup. High-risk coronary artery plaque in asymptomatic patients with type 2 diabetes: clinical risk factors and coronary artery calcium score. Cardiovasc Diabetol. 2021;20(1):164. 10.1186/s12933-021-01350-2 PMC8353743 34372839

[30] Halon DA, Lavi I, Barnett-Griness O, Rubinshtein R, Zafrir B, Azencot M, et al. Plaque morphology as predictor of late plaque events in patients with asymptomatic type 2 diabetes: a long-term observational study. JACC Cardiovasc Imaging. 2019;12(7 Pt 2):1353–63. 10.1016/j.jcmg.2018.02.025 29778864

[31] Xiangli Xu, Yang Hua, Beibei Liu, Fubo Zhou, Lili Wang, Weihong Hou.Association Between Calcification Characteristics of Carotid Atherosclerotic Plaque and Plaque Vulnerability. Ther Clin Risk Manag. 2021; 17:679–690. 10.2147/TCRM.S303485 PMC8257076 34234444

[32] Zeitouni M, Sabouret P, Kerneis M (2021). 2019 ESC/EAS guidelines for management of dyslipidaemia: strengths and limitations. Eur Heart J Cardiovasc Pharmacother.

[33] Wei-Jun YIN, Jing, Ying-Qian ZHANG, Feng TIAN, Tao ZHANG, Shan-Shan ZHOU, Yun-Dai CHEN (2021). Association between non-culprit healed plaque and plaque progression in acute coronary syndrome patients: an optical coherence tomography study. J Geriatr Cardiol.

